# Endothelin-1 Stimulates the Growth of Visceral and Subcutaneous Human Preadipocytes through Similar and Alternative Signaling Pathways via Type A and Type B Endothelin Receptors: Potential Implications for Therapeutic Strategies for Obesity and Metabolic Disorders

**DOI:** 10.7150/ijms.110073

**Published:** 2025-10-01

**Authors:** An-Ci Siao, Yung-Hsi Kao, Chih-Chun Kuo, Hann-Yeh Shyu, Yow-Chii Kuo, Wen-Fang Chiang, Kuo-An Wu, Li-Jane Shih, Po-Jen Hsiao

**Affiliations:** 1 Department of Life Sciences, National Central University, Taoyuan, Taiwan.; 2 Division of Endocrinology, Department of Internal Medicine, Taoyuan Armed Forces General Hospital, Taoyuan, Taiwan.; 3 Division of Neurology, Department of Internal Medicine, Taoyuan Armed Forces General Hospital, Taoyuan, Taiwan.; 4 Division of Gastroenterology, Landseed Hospital, Taoyuan, Taiwan.; 5 Division of Nephrology, Department of Internal Medicine, Taoyuan Armed Forces General Hospital, Taoyuan, Taiwan.; 6 Division of Nephrology, Department of Internal Medicine, Tri-Service General Hospital, National Defense Medical University, Taipei, Taiwan.; 7 Division of Thoracic Medicine, Department of Internal Medicine, Taoyuan Armed Forces General Hospital, Taoyuan, Taiwan.; 8 Department of Medical Laboratory, Taoyuan Armed Forces General Hospital, Taoyuan, Taiwan.; 9 Graduate Institute of Medical Science, National Defense Medical Center, Taipei city, Taiwan.

**Keywords:** endothelin-1, human preadipocytes, white fat cell, AMP-activated protein kinase, obesity, metabolic disease

## Abstract

Endothelin-1 (ET-1), a potent vasoconstrictor, plays multifaceted roles in cellular growth, differentiation, and metabolic regulation. Elevated plasma levels of ET-1 have been observed in obesity, in which ET-1 regulates adipogenesis and the endocrine activity of fat cells. Human white adipocytes are central to energy storage and endocrine regulation. However, relatively little is known about the involvement of the ET-1 signaling pathway in the growth of human white preadipocytes (HWPs). Dysfunction or dysregulation of HWPs may contribute to the development of obesity and associated diseases. Therefore, we investigated the cellular signaling mechanisms in HWPs, focusing on the cellular and functional basis of the actions of ET-1. In this study, signaling protein levels, cell proliferation, and the numbers of visceral and subcutaneous HWPs were measured by immunoblotting and 3-(4,5-dimethylthiazol-2-yl)-2,5-diphenyltetrazolium bromide (MTT), and trypan blue exclusion assays. Both ET type A receptor (ETAR) and ET type B receptor (ETBR) antagonists inhibited the ET-1-induced growth of visceral HWPs and the phosphorylation of AMPK, PKC, and STAT3 in these cells. The ETBR antagonist alone blocked the ET-1-induced phosphorylation of ERK and c-JUN. Pretreatment with specific inhibitors of AMPK, ERK, JNK, STAT3, and PKC prevented ET-1-induced cell proliferation and attenuated the phosphorylation of AMPK, ERK, c-JUN, STAT3, and PKC induced by ET-1. Similar ETAR- and ETBR-dependent and AMPK- and ERK-dependent effects of ET-1 on the growth of primary subcutaneous HWPs were observed. In summary, the transduction of growth-related ET-1 signals in HWPs could occur through similar (e.g., AMPK, PKC, and JAK2/STAT3) or different (e.g., ERK and JNK/c-JUN) pathways. These distinct signaling pathways, along with the optimization of pathway-specific inhibitors, have potential implications for the future management of obesity and metabolic disorders.

## Introduction

Endothelin (ET) is a 21-amino acid peptide hormone that is produced mainly by endothelial cells and plays a key role in maintaining vascular homeostasis 1. There are three members of the ET family: ET-1, ET-2, and ET-3. In particular, ET-1 is produced by airway epithelial cells, fibroblasts, macrophages, neurons in the brain, pancreatic islets, smooth muscle cells, and cardiac myocytes 1-3. With aging, the expression and activity of ET-1 and its receptors may increase, promoting vasoconstriction, vascular stiffness, and endothelial dysfunction. With age, endothelial cells, which line blood vessels, become less responsive to vasodilators such as nitric oxide (NO), shifting the vascular balance toward vasoconstriction and inflammation. This dysregulation contributes to hypertension, a common age-related condition, and atherosclerosis, or the development of plaques in arteries 2-4. The wide distribution of ET-1 in various types of cells, in addition to the presence of two main types of ET receptors, ET type A receptor (ETAR) and ET type B receptor (ETBR), may reflect multiple biological functions of ET-1. As the body ages, the endothelin system becomes dysregulated, contributing to various diseases, particularly cardiovascular and metabolic disorders. For example, ET-1 stimulation of ETAR induces vasoconstrictor tone to contribute to the development and progression of many kinds of human diseases, including hypertension and atherosclerotic vascular disease 5-8. ET-1 levels are significantly elevated in obese individuals. Adipose tissue-derived ET-1 attenuates insulin-mediated antilipolysis in human visceral adipocytes 9.

The cellular effects of ET-1 on the expression of adipocyte genes can be mediated through at least a few pathways: the mitogen-activated protein kinase (MAPK) pathway, the PI3K/AKT pathway, and the Janus kinase 2 (JAK2)/signal transducer and activator of transcription (STAT3) pathway 2, 10-12. The ERK pathway mediates the ET-1-stimulated expression of glucose transporter 1, SOCS3, and resistin. The PI3K/AKT pathway controls ET-1-stimulated glucose transporter 4 (GLUT4) translocation and SOCS3 and resistin expression. The c-Jun N-terminal kinase/c-Jun (JNK/c-JUN) pathway and the JAK2/STAT3 pathway control the ET-1-stimulated expression of SOCS3. In 3T3-L1 adipocytes, ET-1 modulates glucose transporter GLUT1 transcription, the secretion of adiponectin and resistin, and the process of lipolysis. Some mechanisms of action of ET-1 include decreased tyrosine phosphorylation of insulin receptor substrate (IRS)-1 and Gαq/11 and insulin-stimulated glucose transport. Other mechanisms involve dependency on the PI3K/AKT signaling pathway, protein kinase C (PKC), and ERK pathway 13-15. In fat cells, ET-1 regulates cell differentiation, glucose uptake, lipid metabolism, and adipokine secretion. Further studies have indicated that elevated serum ET-1 concentrations are associated with numerous metabolic syndromes, such as obesity, hypertension, uremia, diabetes mellitus, and disorders of the kidney, lung, and liver 16-24.

Recently, ET-1 was reported to regulate murine preadipocyte growth through multiple pathways, including the ETAR, PKC, STAT3, AMPK, c-JUN, ERK, sphingosine kinase, and sphingomyelinase pathways 25. However, ETBR does not mediate the ET-1-stimulated growth of murine white preadipocytes. The ETAR-dependent and ETBR-independent effects of ET-1 may be attributable to increased expression of the ETAR protein and, to a much lesser extent, ETBR in murine preadipocytes. Despite the demonstrations that ET-1 has multiple effects on murine fat cells and stimulates preadipocyte growth via ETAR- but not ETBR-dependent pathways 10-12, 25, it is unknown whether ET-1 affects the growth of human white preadipocytes (HWPs) by modulating the expression of ET receptor (ETR) signaling molecules, particularly ETBR. To date, the biological mechanisms responsible for transducing growth-related ET-1 signals in HWPs remain unclear. Thus, this study was designed to examine the effect of cellular ET-1 signaling on HWPs in relation to the common and distinct ETAR and ETBR signaling pathways. We also summarize the physiological properties of the ET system and its role in the pathophysiology of ET system-activated diseases and discuss potential therapeutic interventions targeting these signaling pathways in obesity and metabolic diseases.

## Materials and Methods

### Chemical reagents

All reagents (e.g., bovine serum albumin and dimethyl sulfoxide) were purchased from Sigma (St. Louis, MO) unless otherwise stated. DMEM, calf serum (CS), trypsin, and protein markers were purchased from GibcoBRL-Invitrogen (New York, NY). All antibodies were purchased from Cell Signaling Technology (Danvers, MA; AMPK, #2793; anti-β-actin, #12620; anti-c-JUN, #9165; anti-pAKT, #4058; anti-pERK, #9101; anti-p38 MAPK, #9212; anti-phospho-AMPK, #2535; anti-phospho-p38 MAPK, #9211; anti-PKCα, #2056; anti-phospho-PKCα/βII, #9375; anti-STAT3, #9139; anti-pSTAT3, #9145; goat anti-rabbit IgG-HRP, #7074; horse anti-mouse IgG-HRP, #7076) or Santa Cruz Biotechnology (Santa Cruz, CA; anti-AKT, sc-8312; anti-ERK1/2, sc-93; anti-JNK, sc-1648; anti-pJNK, sc-6254; anti-pc-JUN, sc-822).

### Cell culture

Primary visceral and subcutaneous HWPs (PromoCell, Germany) were plated at a density of 5,000 cells/cm^2^ in a 10-cm dish according to the manufacturer's instructions. The cells were grown in medium (PromoCell, Germany) supplemented with SupplementMix (final supplement concentrations: 5% v/v fetal calf serum, 0.004 mL/mL endothelial cell growth supplement, 10 ng/mL epidermal growth factor, 1 μg/mL hydrocortisone, and 90 μg/mL heparin) in a humidified atmosphere of 95% air-5% CO_2_ at 37 °C. The medium was replaced every 3-4 days. HWPs were subcultured after they reached confluence, and preadipocytes were passaged before they reached confluence to avoid cell differentiation. The number of passages for HWPs ranged from 2-8.

### Growth stimulation experiments

As described in detail previously 12, subcutaneous HWPs or visceral HWPs (20,000 cells/well) were plated in triplicate wells of a 12-well plate. After incubation for 24 h to allow attachment, the cells were treated with ET-1 (Bachem Co., Bubendorf, Switzerland) dissolved in sterile water containing 5% acetic acid solution. After being incubated with ET-1, the cells were trypsinized and counted on a hemocytometer using the 0.4% trypan blue exclusion method. Cell viability was measured with blue formazan, which is produced from colorless 3-(4,5-dimethylthiazol-2-yl)-2,5-diphenyltetrazolium bromide (MTT) by mitochondrial dehydrogenases, which are active only in live cells. 3T3-L1 preadipocytes were preincubated in 96-well plates at a density of 6 × 10^4^ cells per well for 24 h. The cells were pretreated with the inhibitor BQ610 for 1 h, treated with ET-1 (100 nM) for 48 h, and grown in 0.5 mg/mL MTT at 37 °C. One hour later, 10 μL of solubilization solution was added to each well, and the absorption values were read at 540 nm on an automated SpectraMax 340 (Molecular Devices, Sunnyvale, CA, USA) microplate reader. The data are expressed as the mean percentage of viable cells vs. the control.

### Western blot analysis

Immunoblot analysis was performed as described by Ku et al. 26. Briefly, cells were lysed in Tris buffer containing 50 mM Tris-HCl (pH 7.5), 250 mM NaCl, 5% glycerol, 1 mM EDTA, 0.2 mM EGTA, 233 10 mM NaF, 10 mM sodium pyrophosphate, 1 mM sodium orthovanadate, 0.4% NP-40, 1 mM PMSF, 5-15 mM 2-mercaptoethanol, and proteinase inhibitor cocktail (1 tablet/10 mL). An aliquot of 50-70 μg of total protein from each cell lysate sample was separated by 12.5% sodium dodecyl sulfate‒polyacrylamide gel electrophoresis (SDS‒PAGE) in 2× gel-loading buffer (100 mM Tris-HCl [pH 6.8], 5% β-mercaptoethanol, 0.1% bromophenol blue, 2% SDS, and 10% glycerol). The electrophoresis-separated proteins were transferred onto PVDF membranes (Millipore, Bedford, MA), which were then incubated with a primary antibody at a dilution of 1:1000 (~0.2 μg/mL), followed by incubation with a secondary antibody (e.g., horse anti-mouse immunoglobulin G [IgG] or goat anti-rabbit IgG conjugated to horseradish peroxidase) at a dilution of 1:2000 (~0.2 μg/mL). The immunoblot bands were visualized by incubating the membranes in Western Lightning^TM^ chemiluminescence reagent (Perkin-Elmer Life Science, Boston, MA) for 5 min, followed by exposure to Fuji film for 1-5 min and scanning with a Microtek ScanMaker i800 (Microtek International, Hsinchu, Taiwan). Each band was quantitatively analyzed using ImageJ. The integrated optical density (IOD) was calculated, and the data were normalized to that of β-actin. Protein levels are expressed relative to the control unless otherwise noted.

### Statistical analysis

The data are expressed as the means ± SEs. Unpaired Student's t tests were used to examine differences between the control and ET-1-treated groups. The observed statistical power after the t test ranged from 0.93-1. One-way ANOVA followed by the Student‒Newman‒Keuls multiple-range test was used to examine differences among multiple groups. Differences were considered significant at p < 0.05. To convert discontinuously normalized data to continuous data, the data were logarithmically transformed, and statistical analysis was performed using SigmaStat (Jandel Scientific, Palo Alto, CA). Before each ANOVA, a normality test and homogeneity of variance test were passed. The observed statistical power after ANOVA ranged from 0.95-1.

## Results

### ET-1 stimulated visceral HWP proliferation

**Figure 1:**
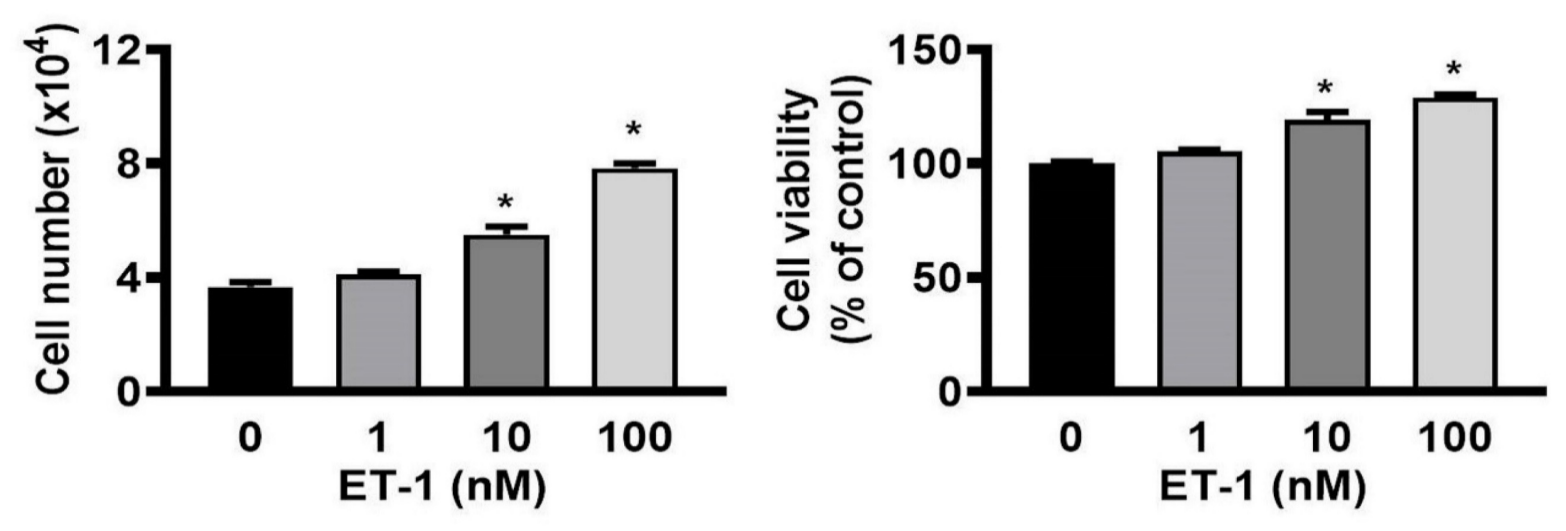
ET-1 stimulated visceral HWP proliferation. Significant changes were noted in the number and viability of visceral HWPs after ET-1 (100 nM) treatment. Pretreatment with either the endothelin type A receptor (ETAR) antagonist BQ610 or the endothelin type B receptor (ETBR) antagonist BQ788 blocked ET-1-mediated alterations in visceral HWP proliferation. All the data are expressed as the mean ± standard error of the mean (SEM) of three independent experiments. *p < 0.05 vs. the control.

To investigate whether the growth of ET-1 affected visceral HWPs, we treated cells with or without 100 nM ET-1 for 48 h. There were significant changes in the number and viability of visceral HWPs after treatment with ET-1 (100 nM). Pretreatment with either the endothelin type A receptor (ETAR) antagonist BQ610 or the endothelin type B receptor (ETBR) antagonist BQ788 blocked ET-1-mediated alterations in visceral HWP proliferation (Fig. 1). These data indicated that ET-1 stimulated visceral HWP growth.

### ET-1 stimulated visceral HWP growth via the ETAR and ETBR pathways

**Figure 2:**
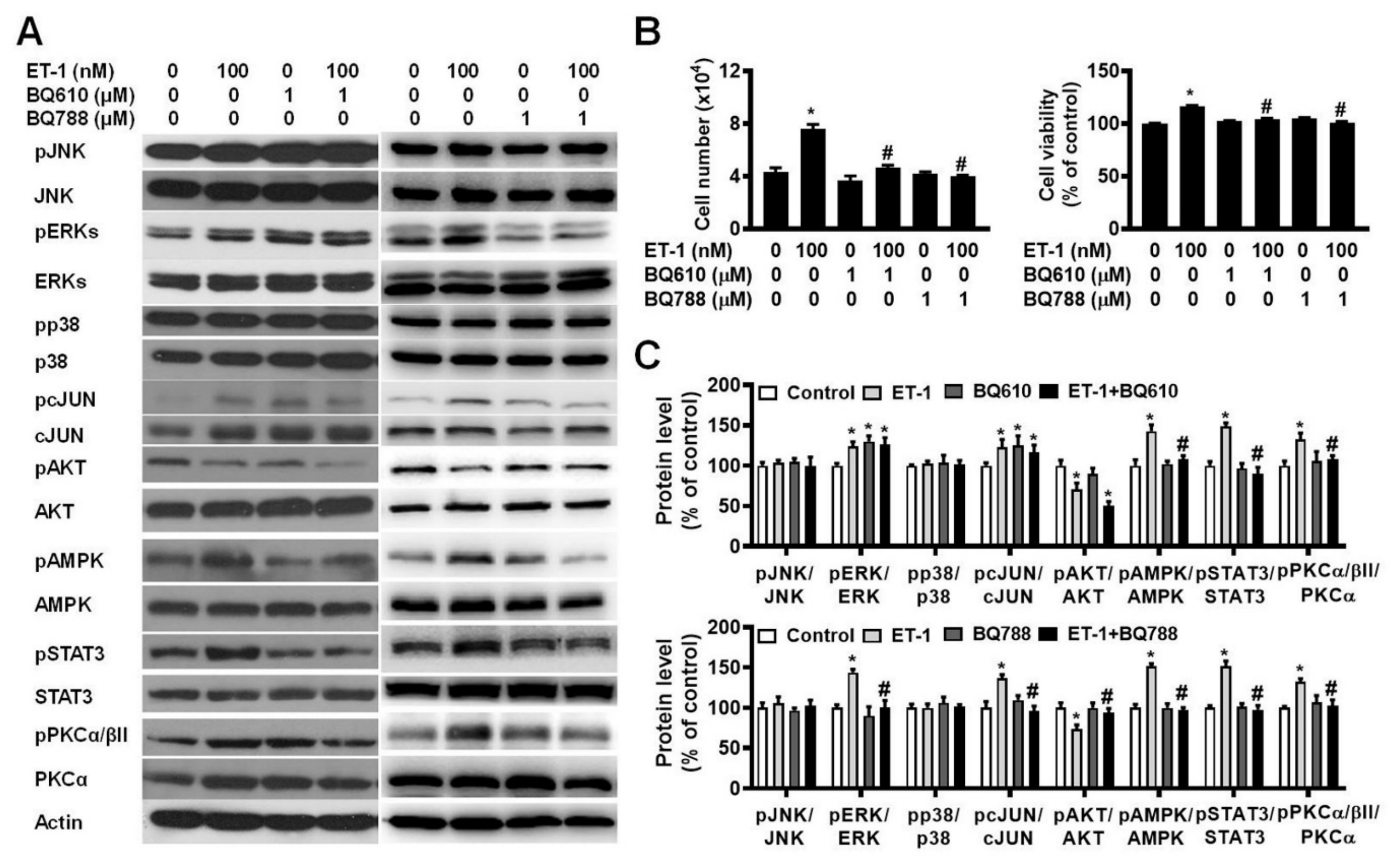
ET-1 stimulated visceral HWP growth via the ETAR and ETBR pathways. We pretreated cells with either BQ610 or BQ788 and then treated them with 100 nM ET-1. According to the cell viability data, pretreatment with BQ610 or BQ788 blocked ET-1-mediated increases in cell viability and cell number: (A) Western blot band. (B) **Si**gnificant changes in cell number and cell viability were noted after BQ610 or BQ788 treatment. (C) ET-1 altered the phosphorylation of the STAT3, AMPK, PKC, ERK, c-JUN, and AKT proteins, and treatment with BQ610 blocked the ET-1-stimulated phosphorylation of the STAT3, AMPK, and PKC proteins in visceral HWPs; BQ788 blocked the ET-1-stimulated phosphorylation of the STAT3, ERK, c-JUN, AMPK, PKC, and AKT proteins in visceral HWPs. All the data are expressed as the mean ± standard error of the mean (SEM) of three independent experiments. *p < 0.05 vs. the control; ^#^*p* < 0.05, ET-1 vs. BQ610 + ET-1, or ET-1 vs. BQ788 + ET-1.

To investigate the effect of ETR on ET-1-mediated changes in visceral HWP growth, we pretreated cells with either BQ610 or BQ788 and then treated them with 100 nM ET-1. According to the cell viability data, pretreatment with BQ610 or BQ788 blocked the ET-1-mediated increases in cell viability and cell number (Fig. 2A).

Previously, we reported that ET-1 stimulates murine 3T3-L1 white preadipocyte growth through multiple kinase pathways, including the AMPK, MAPK, JAK2/STAT3, and PKC pathways, and that these signaling cascades are downstream of ETAR 25. We assessed whether any of these signaling proteins are regulated by ET-1 through the ETAR and ETBR pathways. Indeed, we found that ET-1 altered the phosphorylation of the STAT3, AMPK, PKC, ERK, c-JUN, and AKT proteins and that treatment with BQ610 blocked the ET-1-stimulated phosphorylation of the STAT3, AMPK, and PKC proteins in visceral HWPs (Fig. 2B). Interestingly, pretreatment with BQ788 blocked the ET-1-stimulated phosphorylation of the STAT3, ERK, c-JUN, AMPK, PKC, and AKT proteins in visceral HWPs (Fig. 2C).

### ET-1 stimulated visceral HWP growth through the ERK pathway

**Figure 3:**
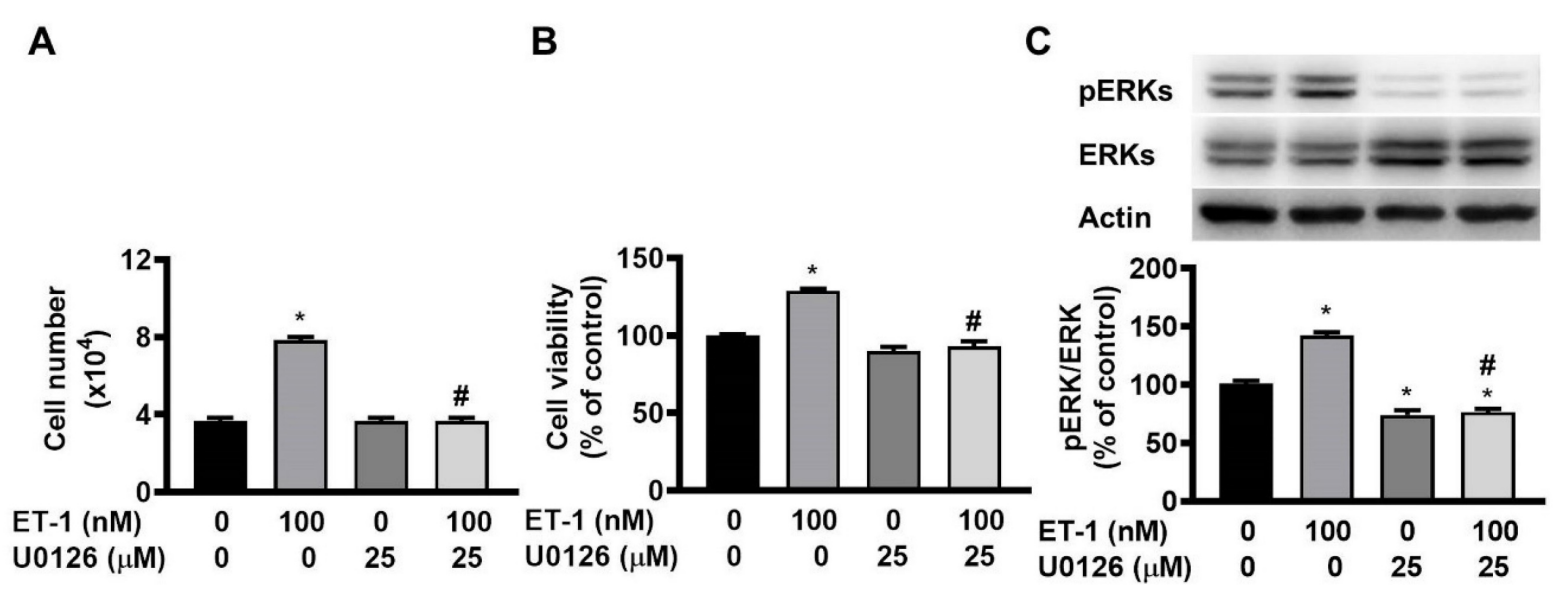
ET-1 stimulated visceral HWP growth through the ERK pathway. The ERK pathway mediates the ET-1-stimulated expression of glucose transporter 1, SOCS3, and resistin. We pretreated cells with the ERK-specific inhibitor U0126 and then incubated them with ET-1 for 48 h. U0126 prevented the ET-1-induced increases in both the number and viability of HWPs (**A** and** B**). Western blot analysis indicated that U0126 prevented the ET-1-induced increase in ERK phosphorylation (**C**). All the data are expressed as the mean ± standard error of the mean (SEM) of three independent experiments. *p < 0.05 vs. the control; ^#^*p* < 0.05, ET-1 vs. U0126 + ET-1.

The ERK pathway mediates the ET-1-stimulated expression of glucose transporter 1, SOCS3, and resistin 12, 13, 15. To investigate the effect of ERK on ET-1-mediated visceral HWP growth, we pretreated cells with the ERK-specific inhibitor U0126 and then incubated them with ET-1. After 48 h of incubation with ET-1, we found that U0126 prevented the ET-1-induced increases in both the number and viability of HWPs (Fig. 3A and B). Additionally, U0126 prevented the ET-1-induced increase in ERK phosphorylation (Fig. 3C).

### ET-1 stimulated visceral HWP growth through the JNK/c-JUN pathway but not the p38 MAPK pathway

**Figure 4:**
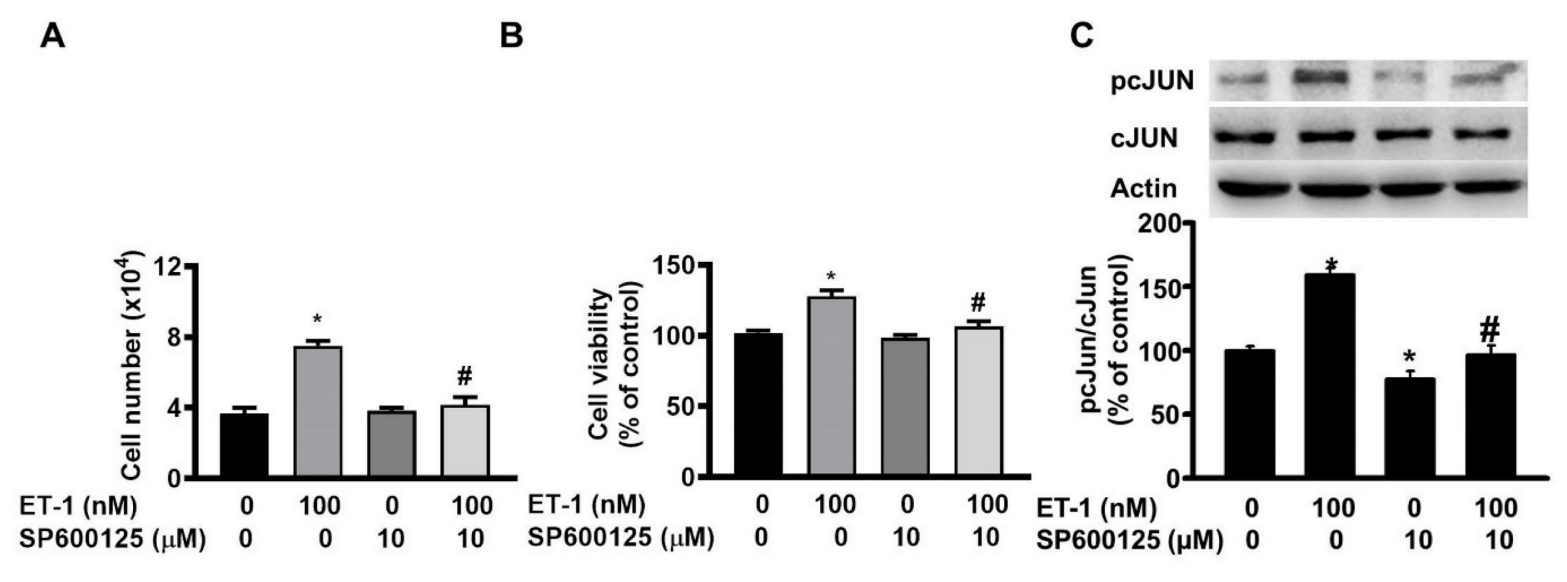
ET-1 stimulated visceral HWP growth through the JNK/c-JUN pathway. We pretreated cells with the JNK/c-JUN-specific inhibitor SP600125 for 1 h and then incubated them with ET-1 for an additional 48 h. After incubation, SP600125 prevented the ET-1-induced increases in both the number and viability of HWPs (**A** and **B**). Western blot analysis revealed that SP600125 prevented the ET-1-induced increase in c-JUN phosphorylation (**C**). All the data are expressed as the mean ± standard error of the mean (SEM) of three independent experiments. *p < 0.05 vs. the control: ^#^*p* < 0.05, ET-1 vs. SP600125 + ET-1.

**Figure 5:**
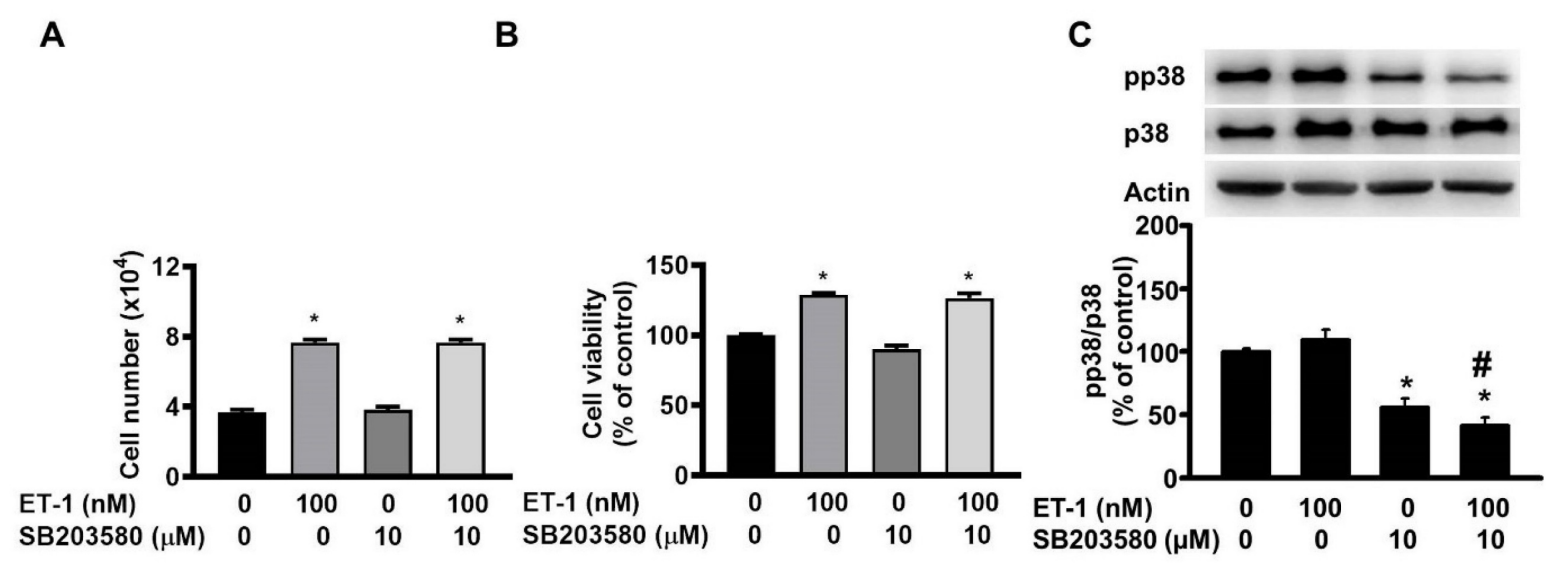
Pretreatment with the p38 MAPK-specific inhibitor SB203580 had no effect on the ET-1-induced increase in the number or viability of HWPs. All the data are expressed as the mean ± standard error of the mean (SEM) of three independent experiments. *p < 0.05 vs. the control; ^#^*p* < 0.05, ET-1 vs. SB203580 + ET-1.

The JNK/c-JUN-mediated pathway controls the ET-1-stimulated proliferation of murine 3T3-L1 preadipocytes 25. To investigate the effect of JNK/c-JUN on ET-1-mediated visceral HWP growth, we pretreated cells with the JNK/c-JUN-specific inhibitor SP600125 for 1 h and then incubated them with ET-1 for an additional 48 h. After incubation, we found that SP600125 prevented the ET-1-induced increases in both the number and viability of HWPs (Fig. 4A and B). Western blot analysis revealed that SP600125 prevented the ET-1-induced increase in c-JUN phosphorylation (Fig. 4C). However, pretreatment with the p38 MAPK-specific inhibitor SB203580 had no effect on the ET-1-induced increase in the number or viability of HWPs (Fig. 5A and B).

### ET-1 stimulated visceral HWP growth through the JAK2/STAT3 pathway

**Figure 6:**
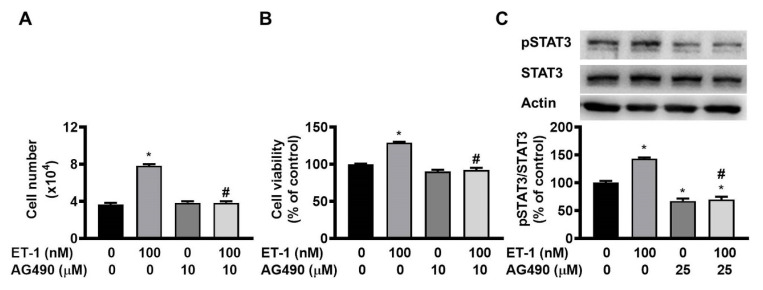
ET-1 stimulated visceral HWP growth through the JAK2/STAT3 pathway. We pretreated cells with the JAK2/STAT3-specific inhibitor AG490 for 1 h and then incubated them with ET-1 for an additional 48 h. AG490 prevented ET-1-induced increases in both the number and viability of HWPs (**A** and **B**). Western blot analysis revealed that AG490 prevented ET-1-stimulated STAT3 phosphorylation (**C**). All the data are expressed as the mean ± standard error of the mean (SEM) of three independent experiments. *p < 0.05 vs. the control; ^#^*p* < 0.05, ET-1 vs. AG490 + ET-1.

The JAK2/STAT3 pathway controls the ET-1-stimulated expression of SOCS3 12. To investigate the effect of JAK2/STAT3 on ET-1-mediated visceral HWP growth, we pretreated cells with the JAK2/STAT3-specific inhibitor AG490 for 1 h and then incubated them with ET-1 for an additional 48 h. We found that AG490 prevented the ET-1-induced increases in both the number and viability of HWPs (Fig. 6A and B). Western blot analysis revealed that AG490 prevented ET-1-stimulated STAT3 phosphorylation (Fig. 6C).

### ET-1 stimulated visceral HWP growth through the AMPK pathway

**Figure 7:**
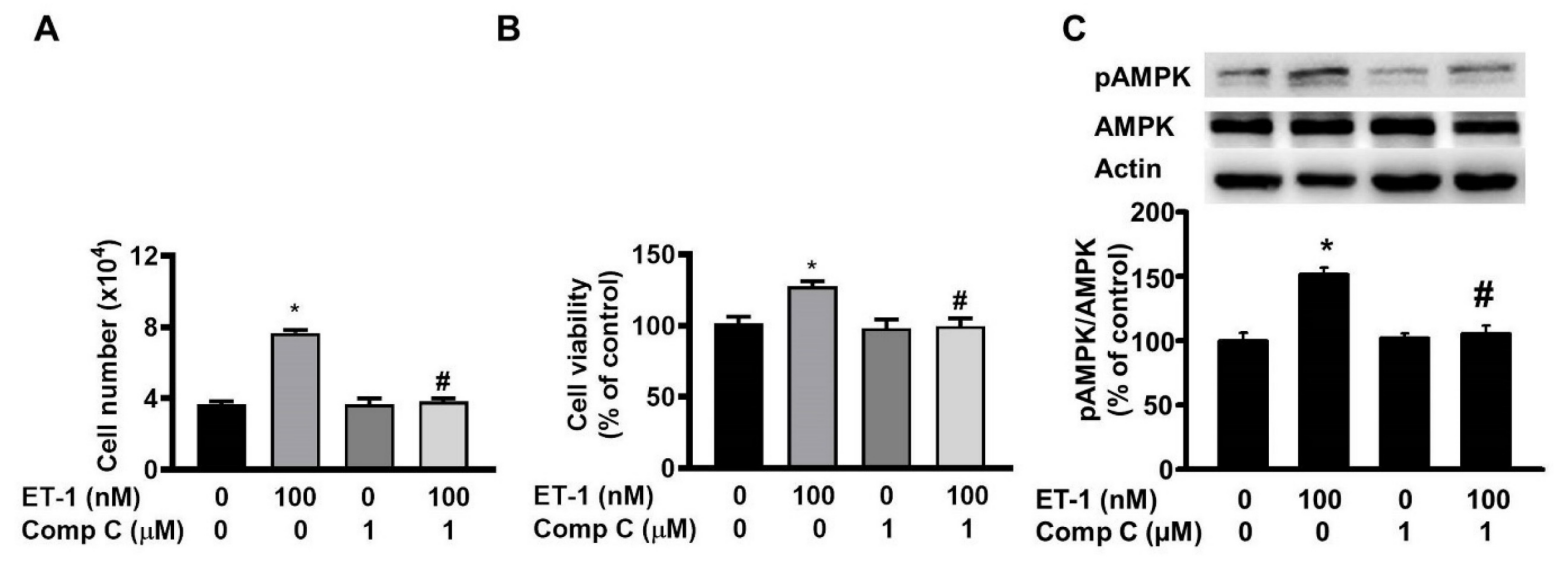
ET-1 stimulated visceral HWP growth through the AMPK pathway. We pretreated cells with the AMPK-specific inhibitor compound C for 1 h and then incubated them with ET-1. Compound C prevented ET-1-induced increases in both the number and viability of HWPs (**A** and **B**). Western blot analysis indicated that compound C prevented the ET-1-induced increase in AMPK phosphorylation (**C**). All the data are expressed as the mean ± standard error of the mean (SEM) of three independent experiments. *p < 0.05 vs. the control; ^#^*p* < 0.05, ET-1 vs. compound C + ET-1.

AMP-activated protein kinase (AMPK) can regulate cell growth and death. To investigate the effect of AMPK on ET-1-stimulated visceral HWP growth, we pretreated cells with the AMPK-specific inhibitor compound C for 1 h and then incubated them with ET-1. We found that compound C prevented ET-1-induced increases in both the number and viability of HWPs (Fig. 7A and B). Western blot analysis indicated that compound C prevented the ET-1-induced increase in AMPK phosphorylation (Fig. 7C).

### ET-1 stimulated visceral HWP growth through the PKC pathway

**Figure 8:**
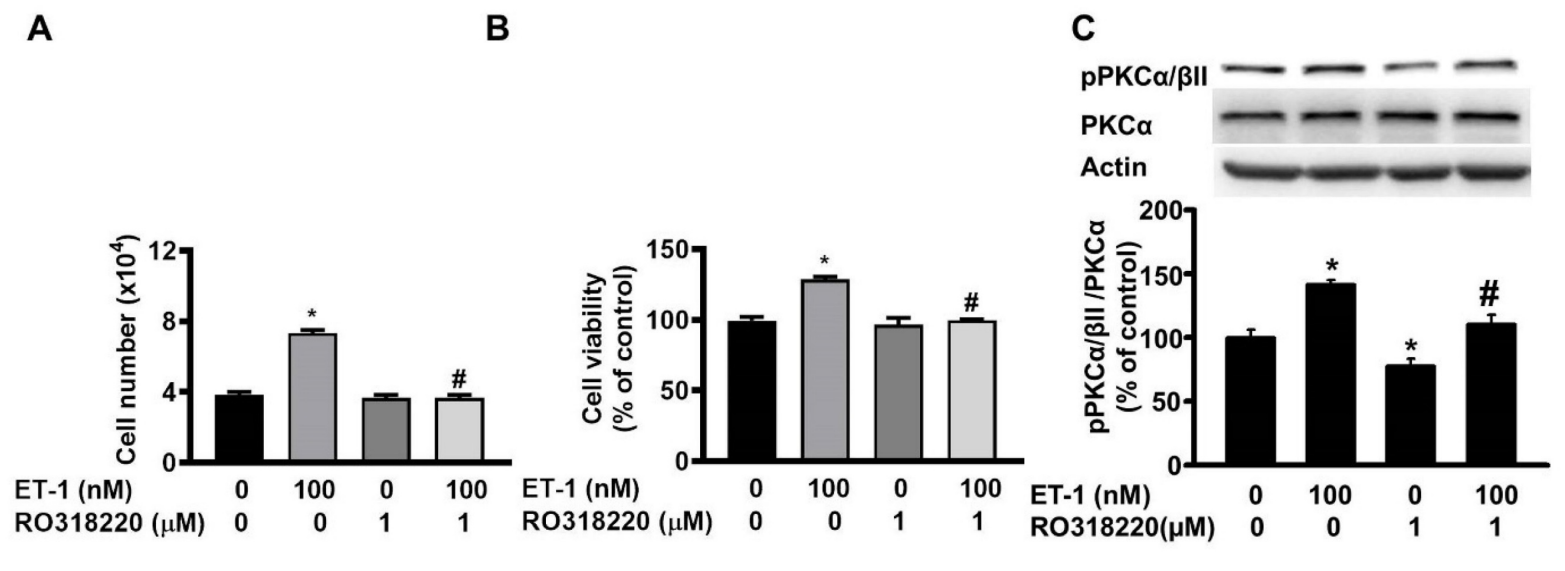
ET-1 stimulated visceral HWP growth through the PKC pathway. We pretreated cells with the PKC-specific inhibitor Ro318220 for 1 h and then incubated them with ET-1 for 48 h. Ro318220 prevented ET-1-induced increases in both the number and viability of HWPs (**A** and** B**). Western blot analysis indicated that Ro318220 prevented ET-1-stimulated PKC phosphorylation (**C**). All the data are expressed as the mean ± standard error of the mean (SEM) of three independent experiments. *p < 0.05 vs. the control; ^#^*p* < 0.05, ET-1 vs. Ro318220 + ET-1.

The protein kinase C (PKC) pathway controls ET-1-induced preadipocyte growth 25. To investigate the effect of PKC on ET-1-mediated visceral HWP growth, we pretreated cells with the PKC-specific inhibitor Ro318220 for 1 h and then incubated them with ET-1. After 48 h of incubation with ET-1, we found that Ro318220 prevented ET-1-induced increases in both the number and viability of HWPs (Fig. 8A and B). Western blot analysis indicated that Ro318220 prevented ET-1-stimulated PKC phosphorylation (Fig. 8C).

### Different effects of various endothelin hormones on visceral HWPs

**Figure 9:**
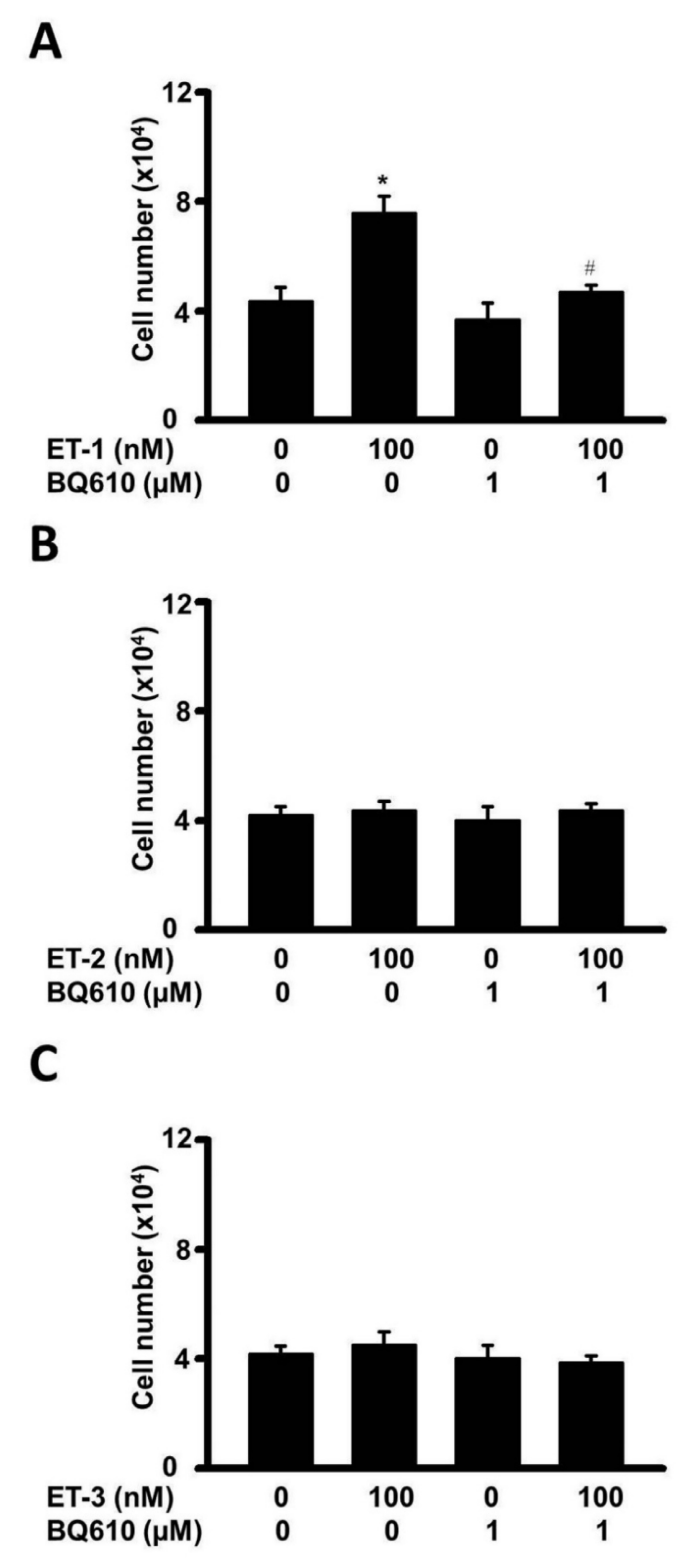
Different effects of various endothelin hormones on visceral HWPs. Changes in HWP proliferation were assessed by the MTT assay. Indeed, ET-1 (**A**) increased the proliferation of visceral HWPs. Interestingly, neither ET-2 (**B**) nor ET-3 (**C**) altered the growth of HWPs.

The endothelin family contains ET-1, ET-2, and ET-3, which are differentially distributed in tissues and have different receptor binding affinities in non-fat cells, reflecting their possibly differential functions in regulating cellular processes 1, 4. To compare the effects of ET-1, ET-2, and ET-3 on the growth of human visceral white preadipocytes, cells were treated with different types of endothelin, and changes in preadipocyte proliferation were assessed by the MTT assay. Indeed, ET-1 was found to increase the proliferation of visceral HWPs. Interestingly, neither ET-2 nor ET-3 altered the growth of HWPs. These data indicated an ET type-dependent effect (Fig. 9).

### ET-1 stimulated subcutaneous HWP growth via the ETAR and ETBR pathways

**Figure 10:**
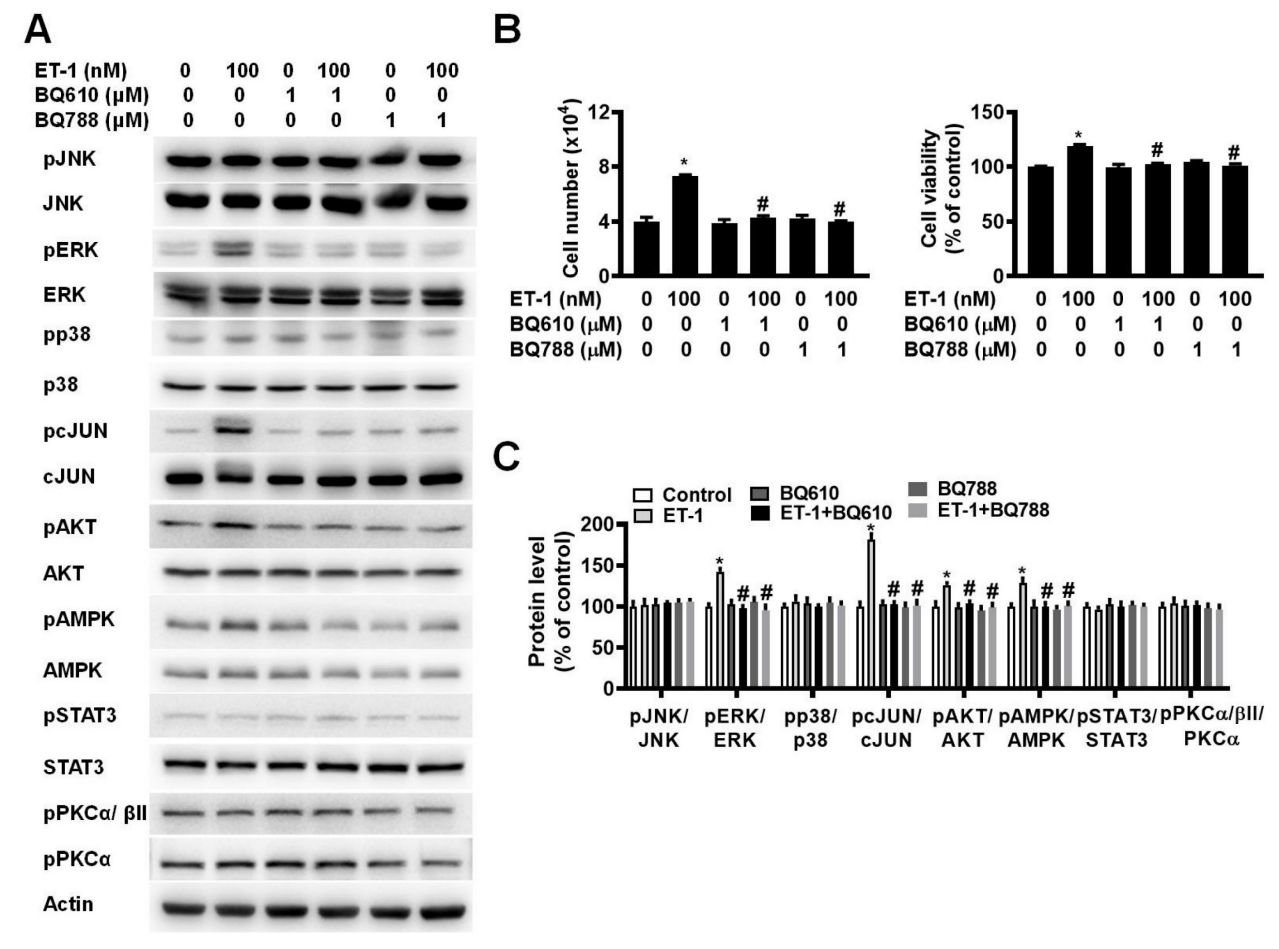
ET-1 stimulated subcutaneous HWP growth via the ETAR and ETBR pathways. We pretreated cells with either BQ610 or BQ788 and then treated them with 100 nM ET-1. According to the cell viability data, pretreatment with BQ610 or BQ788 blocked ET-1-mediated increases in cell viability and cell number: (A) Western blot bands. (B) Significant changes in cell number and cell viability were noted after BQ610 or BQ788 treatment. (C)** E**T-1 altered the phosphorylation of the AMPK, ERK, c-JUN, and AKT proteins, and treatment with either BQ610 or BQ788 blocked the ET-1-stimulated phosphorylation of the ERK, c-JUN, AMPK, and AKT proteins in subcutaneous HWPs. All the data are expressed as the mean ± standard error of the mean (SEM) of three independent experiments. *p < 0.05 vs. the control; ^#^*p* < 0.05, ET-1 vs. BQ610 + ET-1, or ET-1 vs. BQ788 + ET-1.

**Figure 11:**
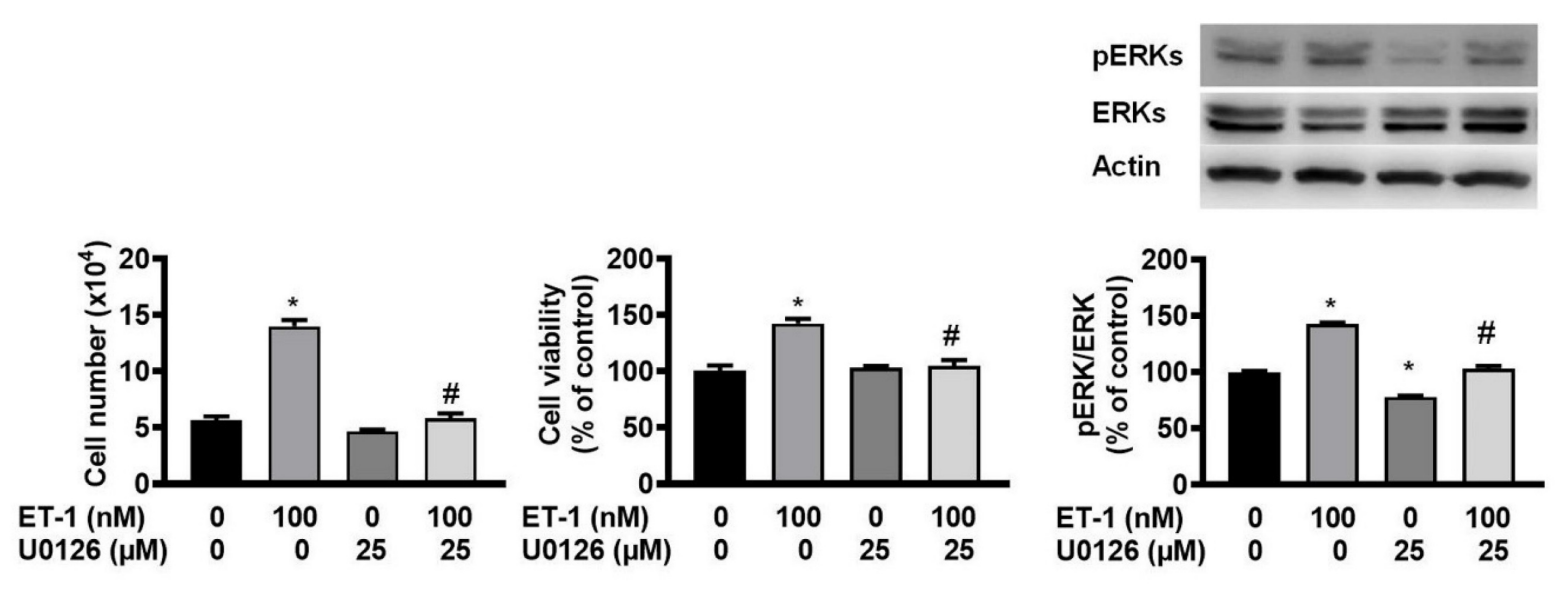
Pretreatment of subcutaneous HWPs with compound C for 1 h blocked the ET-1-induced increase in pAMPK protein levels. All the data are expressed as the mean ± standard error of the mean (SEM) of three independent experiments. *p < 0.05 vs. the control; ^#^*p* < 0.05, ET-1 vs. Comp C + ET-1.

**Figure 12:**
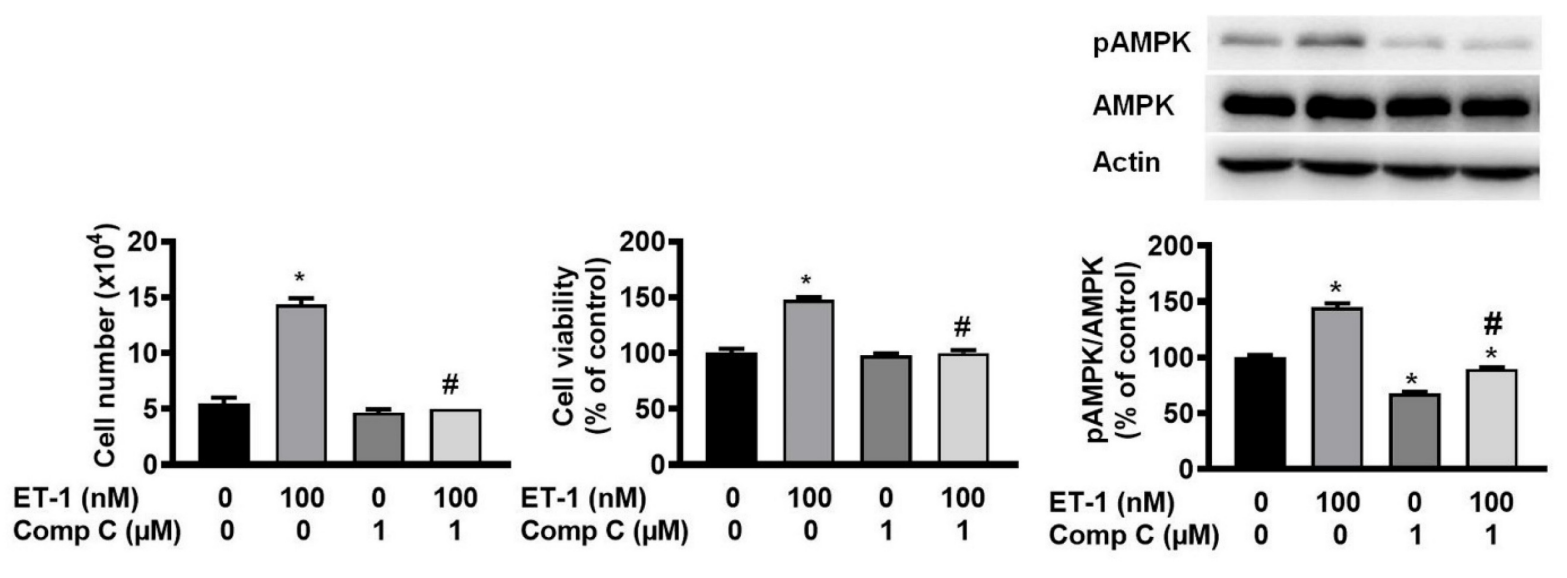
Pretreatment of subcutaneous HWPs with U0126 for 1 h blocked the ET-1-induced increase in pERK protein levels. All the data are expressed as the mean ± standard error of the mean (SEM) of three independent experiments. *p < 0.05 vs. the control; ^#^*p* < 0.05, ET-1 vs. U0126 + ET-1.

In support of this ETAR- and ETBR-dependent effect of ET-1, we found that treatment with 100 nM ET-1 for 48 h induced a 70% increase in cell number and a 20% increase in cell proliferation in primary subcutaneous HWPs (Fig. 10). Pretreatment of these subcutaneous HWPs with either 1 µM BQ610 or 1 µM BQ788 for 1 h prevented the ET-1-induced increases in cell number and mitogenesis (Fig. 10B). Neither BQ610 nor BQ788 alone had a significant effect on cell number or cell proliferation. The changes in subcutaneous HWPs were similar to those observed for visceral HWPs, supporting the notion that ET-1 stimulates HWP growth through ETAR- and ETBR-dependent effects. Interestingly, pretreatment with BQ610 or BQ788 blocked the ET-induced increases in the phosphorylation levels of ERK, c-JUN, AKT, and AMPK proteins in subcutaneous HWPs (Fig. 10A). ET-1, BQ610, and BQ788 did not alter the levels of pJNK, pp38, pSTAT3, or pPKCα/βII. We also observed that pretreatment of subcutaneous HWPs with either compound C or U0126 for 1 h blocked the ET-1-induced increases in cell number, cell proliferation, and pAMPK and pERK protein levels (Figs. 11 and 12), further supporting the AMPK- and ERK-dependent effects of ET-1.

## Discussion

### The ET system in HWPs

In the past, research has predominantly focused on the role of ET-1 signaling via ETAR in adipocytes, with little emphasis on the role of ETBR. While the role of ET-1 signaling in controlling murine preadipocyte is was primarily mediated through the ETAR rather than the ETBR pathway 25, the effects of ET-1 signaling pathways on the growth of visceral HWPs via both ETAR and ETBR were demonstrated in this study. The ERK-mediated pathway could be downstream of ETAR signaling and transduce growth-related ET-1 signals to murine preadipocytes but not to human visceral preadipocytes; however, ET-1 signaling controls visceral HWP growth in a manner mediated by ETBR signaling. These discoveries suggest that both ETAR and ETBR may act independently to mediate the stimulatory effect of ET-1 on the growth of human preadipocytes through different downstream signaling molecules. Notably, fat cells in rodents express greater ETAR mRNA levels and no or lower levels of ETBR mRNA, whereas human fat cells equally express high ETAR and ETBR mRNA levels [27; Supplemental Figure 1]. This may explain the ETBR-independent effects of ET-1 in murine preadipocytes and the ETBR-dependent effects of ET-1 in visceral HWPs, in addition to supporting the species-specific differences in adipocytes and adipose tissues between mice and humans 28.

The plasma concentrations of ET-1, ET-2, and ET-3 are typically approximately 5, 0.9, and 0.3 pM, respectively, and they are widely distributed in human tissues 27. However, ET-1, ET-2, and ET-3 are produced mainly by endothelial cells, ovarian granulosa cells and the intestinal epithelium, and the brain, respectively 27. The order ET-1, ET-2, and ET-3 in terms of binding affinity for ETAR is ET-1 > ET-2 >> ET-3 (100-fold lower); however, ET-1, ET-2, and ET-3 have similar affinities for ETBR 27. All of these observations reflect possible distinct functions of ET-1, ET-2, and ET-3 in particular types of cells. Indeed, ET-1 and ET-3, but not ET-2, were found to stimulate murine 3T3-L1 preadipocyte growth through different pathways associated with ETAR 25. However, no studies have demonstrated whether ET-1, ET-2, or ET-3 influence HWP growth. Thus, our study is the first to compare the ability of different endothelin family members to regulate the growth of HWPs. By comparing the effects of ET-1, ET-2, and ET-3 on the growth of visceral HWPs, we found that ET-1 significantly increased the number and viability of HWPs and that neither ET-2 nor ET-3 altered visceral HWP proliferation. The various effects of different ET subtypes on the growth of visceral HWPs may be explained by differences in binding affinities for different receptor types and differences in the levels of splicing mutants, posttranslational modifications, and downstream signaling molecules for both receptors 27. This notion is indirectly supported by the findings related to ET-1, as ET-1 was found to have the highest binding affinity for ETAR among ET family members, and the finding that ETAR protein expression but not ETBR protein expression is higher in the adipose tissues of obese human subjects than in those of lean subjects 27, 29. In addition, Davenport et al. (2016) reported that splicing variants and translational modifications of ETAR and ETBR can alter receptor functions 27.

Although both ETAR and ETBR are G protein-coupling receptors traditionally known to activate Gq signaling, ETBR has been linked to other Gs or Gi proteins and other signaling systems (e.g., the MAPK/ERK signaling pathway) 27. Further inhibition of ET-1/ETAR/Gq signaling in human adipose precursor cells stimulates thermogenic adipocyte differentiation, whereas activation of ET-3/ETBR/ERK promotes browning of HWPs 27, 29-30. In parallel, different ET-1 and ET-3 signals in 3T3-L1 preadipocytes mediate the growth process 25, 31. Notably, ETAR and ETBR are monomeric proteins, but they can form homodimers or heterodimers to mediate Ca^2+^ signaling in human embryonic kidney 293 cells 27, 29. Accordingly, determining whether ET-1, ET-2, and ET-3 affect the dimerization of ETAR or ETBR in HWPs and thereby lead to their different effects on the growth process requires further study. The lack of effect of ET-3 on human preadipocyte growth was inconsistent with our previous report 31 on its stimulatory effect on murine preadipocyte growth. This inconsistency may be attributable to species specificity, as indicated above 28. Taken together, these findings demonstrate whether ET-1, ET-2, and ET-3 differentially bind to both ETAR and ETBR in HWPs and alter the number, dimerization, and signaling molecules of each receptor, which should help clarify the different effects of ET family members in HWPs.

We attempted to identify the downstream signaling transducers of ET-1 that are involved in stimulating visceral HWP growth. In this study, we showed that ET-1 altered the phosphorylation of the STAT3, ERK1/2, AMPK, c-JUN, and PKC proteins. Pretreatment with specific inhibitors of ETAR, i.e., BQ610, prevented the ET-1-stimulated phosphorylation of the STAT3, AMPK, and PKC proteins. However, BQ610 did not inhibit the ET-1-induced changes in ERK1/2 or c-JUN phosphorylation. These findings differed from those reported for the signaling proteins of ET-1 that stimulate 3T3-L1 preadipocyte growth 25. This difference may be explained by species specificity because ETAR is more highly expressed than ETBR in 3T3-L1 fat cells and because both receptors are almost equally expressed in human fat cells 25, 27, 29, 32. Our findings revealed that ETAR, but not ETBR, was expressed in 3T3-L1 white, C3H10T1/2 white, and HIB1B brown fat cells (Supplemental Figure 1). In addition, Davenport et al. (2016) reported that ETAR and ETBR are expressed equally in adipocytes, white adipose tissue, and brown adipose tissue 27. Moreover, pretreatment with the ETBR antagonist BQ788 prevented ET-1-mediated visceral HWP growth and blocked ET-1-induced increases in the phosphorylation of the AMPK, STAT3, ERK1/2, c-JUN, and PKC proteins. These observations suggest that the downstream ETAR and ETBR signaling molecules that transduce ET-1 growth signals in visceral HWPs can be similar (e.g., AMPK, PKC, and JAK2/STAT3) or different (e.g., ERK and JNK/c-JUN). However, understanding how these receptor subtypes differentially regulate key pathways in visceral HWPs requires further investigation. Interestingly, the results of this study indicated that downstream ETAR and ETBR signaling molecules transduce growth-related ET-1 signals in subcutaneous HWPs, most likely in a similar way as the ERK, c-JUN, AMPK, and AKT pathways. This is because pretreatment with either BQ610 or BQ788 blocked the ET-1-induced increases in the phosphorylation of these proteins.

This study suggests that the molecules targeted by ETAR and ETBR signaling to transduce growth-related ET-1 signals between visceral and subcutaneous HWPs may be similar or different. Similar ETAR signaling is dependent on AMPK, as pretreatment with BQ610 suppressed the ET-1-induced increase in AMPK phosphorylation in both types of HWPs. Similar ETBR signaling is dependent on AMPK, ERK1/2, and c-JUN cascades, as pretreatment with BQ788 blocked the ET-1-induced increase in the phosphorylation of AMPK, ERK1/2, and c-JUN proteins. However, the ERK-mediated pathway regulates the stimulatory effect of ETAR signaling on the growth of subcutaneous HWPs but not visceral HWPs, as pretreatment with BQ610 blocked ET-1-stimulated ERK phosphorylation in the former but not in the latter. The JAK2/STAT3-mediated pathway and the PKC-mediated pathway regulated the effect of ET-1 on the growth of visceral but not subcutaneous HWPs because pretreatment with either BQ610 or BQ788 blocked ET-1-induced increases in pSTAT3 and pPKCα/βII levels in visceral but not subcutaneous HWPs. It is worthwhile to explore whether any of these ETAR and ETBR signaling pathways observed in vitro also mediate the effect of ET-1 on the growth of HWPs in different regions in vivo.

### Roles of ET-1 in obesity and metabolic disorders

The effects of ET-1 on obesity and metabolic disorders have been reported 27, 29, 35. In particular, plasma ET-1 levels are elevated in obese individuals, and this upregulation is associated with increased adipose tissue inflammation, insulin resistance, diabetes mellitus, cardiovascular complications, and hypertension 27, 29, 32-35. Although ET-1 can impair adipogenesis by inhibiting the differentiation of preadipocytes into adipocytes and reducing lipid accumulation, potentially contributing to dysfunctional adipose tissue remodeling in obesity, ET-1 infusion can induce fat cell hyperplasia 29, 36, 37. Physiologically, ET-1 can regulate basal vascular tone and metabolic function via different receptors on various tissues, including the endothelium, vascular smooth muscle cells, adipocytes, and hepatocytes 1-6, 12-13, 27, 29, 32-39. In the vasculature, ET-1 influences blood pressure through vasoconstriction and plays a crucial role in regulating basal vascular tone. ET-1 indirectly affects cardiac muscle by modulating coronary artery tone and directly affects muscle and cardiac output 34. ET-1 also stimulates the contraction of smooth muscle cells in the airway and gastrointestinal tract 27. In the kidneys, ET-1 causes the contraction of mesangial cells to modulate the glomerular filtration rate and sodium and water reabsorption 40. In addition, ET-1 can modulate adipose tissue inflammation, vascular permeability, insulin resistance, and immune responses through the modulation of adipokine secretion and insulin signaling pathways 1, 12, 27, 29, 34-39, 41. Given that obesity is characterized by adipocyte hypertrophy and hyperplasia in association with increased infiltration of immune cells and elevated secretion of proinflammatory cytokines and that the processes of adipocyte mitogenesis, adipogenesis, and adipokine secretion can be regulated by the ET-1/ET receptor system 12, 25, 27, 29, 36-39, elucidating the role of ET-1 signaling in adipose tissue expansion and dysfunction provides insights into the pathogenesis of obesity- and-adipokine-related metabolic disorders 29, 35.

### Potential therapeutic strategies targeting ET-1 signaling pathways

Although the effects of selective ETAR antagonists and dual ETAR and ETBR antagonists have been clinically tested in several pathophysiological conditions, including hypertension, heart failure, and diabetic nephropathy, few studies have reported on the efficacy of targeting the endothelin system and downstream signaling molecules as a therapeutic strategy for obesity-related complications 27, 29, 41-43. Treatment with the ETAR antagonist atrasentan (10 mg/kg/day) or the mixed ETAR/ETBR antagonist bosentan (100 mg/kg/day) for 4 weeks was able to lower body weight, reduce adipose tissue inflammation, and improve glucose tolerance in mice with systemic lupus 41. Both atrasentan and bosentan therapy could also decrease glomerulosclerosis, and atrasentan could lower renal T-cell infiltration, reflecting the possible efficacy of ET receptor antagonists designed to slow the progression of chronic kidney disease (CKD) and diabetic kidney disease 41-43. In addition, ET-1 infusion can induce fat cell hyperplasia and resistin expression, and treatment with an ETAR antagonist blocks ET-1-mediated mitogenesis and adipokine expression and secretion in murine primary and secondary preadipocytes 12, 25, 36, 39. Our present study showing that both ETAR and ETBR antagonists could block ET-1 growth signals in visceral and subcutaneous HWPs supports the possible use of these antagonists in controlling the number of fat cells and their potential applications in fat cell-associated obesity and other metabolic disorders.

The downstream signaling molecules of ETR in fat cells or in association with obesity-related complications have been targeted 29, 41, 44-48. STAT3 inhibitors, such as stattic or napabucasin, can suppress ET-1-mediated adipogenesis and mitigate inflammation-driven metabolic dysfunction 45, 46, and AG490 blocks the ET-1-induced increases in the mRNA and protein levels of adipocyte resistin and suppressor of cytokine signaling 3 (SOCS-3) 12-13. Specific inhibitors of MEK, such as trametinib or PD98059, can suppress increased ET-1-mediated vasoconstriction after organ culture in ocular arteries 48 and inhibit the ET-1-stimulated expression of the resistin and SOCS-3 genes in adipocytes 12, 13. PKC inhibitors, such as ruboxistaurin or Ro318220, have shown promise in other disease contexts, including diabetic complications 47, in addition to inhibiting the ET-1-stimulated growth of murine preadipocytes 25. AMPK plays a pivotal role in cellular energy homeostasis, and a previous study showed that treatment with specific inhibitors of AMPK, such as compound C, suppresses the ET-1-, IGF-I-, and IGF-II-mediated growth of murine preadipocytes 25, 44. In support of the utilization of these specific inhibitors of downstream ETR signaling molecules as possible therapies for fat cell-related disease, the results of the present study revealed that ET-1 activated AMPK, PKC, JAK2/STAT3, and MEK1/ERK to stimulate preadipocyte growth, depending on the particular type of ETR, the location of preadipocytes, and kinase signaling cascades. Given the interconnected nature of these signaling pathways 25, a multitarget therapeutic approach may be most effective. Combination therapies that simultaneously modulate AMPK, ERK, PKC, and STAT3 pathways could synergistically inhibit ET-1-mediated fat cell activity, obesity, and obesity-related complications while improving metabolic health. Notably, other studies have shown that AMPK promotes lipid oxidation and glucose uptake in muscle and adipose tissues 49. Accordingly, selective modulation of AMPK could provide dual benefits: inhibition of preadipocyte proliferation combined with enhanced energy expenditure. As there are many PKC isoforms 50, the development of isoform-specific PKC inhibitors could enhance therapeutic precision and minimize the adverse effects of ET-1 signaling. As resistin/STAT3/SOCS3 are central players in inflammation and metabolism 12-13, 45, 46, their activation by ET-1 may link preadipocyte growth and adipokine signaling to inflammatory and metabolic pathways. Thus, combining STAT3 inhibitors with antiadipokine and anti-inflammatory agents might further enhance therapeutic outcomes. To our knowledge, the MEK1/ERK and JNK/c-JUN pathways are critical regulators of cell proliferation and differentiation 25, 36, 37. Our study indicates that the activation of these genes by ET-1 may underscore their roles in preadipocyte growth and adipose tissue expansion. However, given the widespread involvement of these kinase pathways in other physiological processes, careful consideration of off-target effects and tissue-specific delivery systems is essential. Advances in nanotechnology and drug delivery systems could enable targeted interventions, reducing systemic side effects. Pharmacological strategies should be complemented by lifestyle interventions, including diet and exercise, which naturally modulate these pathways. For instance, physical activity activates AMPK and reduces inflammation, counteracting the effects of ET-1. Integrating lifestyle changes with targeted therapies may provide a holistic approach to managing obesity and metabolic disorders. Although targeting ET-1-stimulated signaling pathways may offer a promising therapeutic strategy for fat cell-related metabolic disorders, clinical trials or extended mechanistic analyses for optimizing pathway-specific inhibitors and developing integrative treatment regimens to achieve sustainable metabolic health improvements still require further thorough studies.

ET-1 may have multiple direct or indirect biological effects on adipocytes, including inflammation, metabolic alterations, energy utilization, and phenotype switching 12, 13, 25, 27, 29, 36-39, 41. ET-1 clearly stimulates the expression of the resistin and SOCS3 genes in 3T3-L1 adipocytes, both of which can regulate inflammatory processes and insulin function, and SOCS3 acts as a downstream signaling molecule of resistin and STAT3 12, 13. Although ET-1 was reported to inhibit the differentiation of murine and human preadipocytes to adipocytes, it was found to increase the proliferation of murine preadipocytes both in vitro and in vivo 25, 29, 36, 37. ET-1 also stimulates cell growth in murine primary preadipocytes 25. In line with the findings of this study, we observed that ET-1 stimulated the proliferation of visceral and subcutaneous HWPs. Notably, compared with those in healthy lean subjects, the levels of ET-1 in obese patients are generally elevated 27, 29. In addition, treatment with ET-1 decreased the expression and secretion of the hormone adiponectin, an adipokine that is involved in regulating glucose levels and fatty acid breakdown, in 3T3-L1 adipocytes 39. Together with these findings, the present finding that ET-1 signals affect HWP growth may increase the clinical applicability of ET-1.

ET-1 has numerous biological effects in vitro, and its effects are generally observed in the range of 1-100 nM 27. In vivo, the plasma concentration of ET-1 generally reported in animals and humans is approximately 5 pM. However, the effectiveness of ET-1 depends on the physiological and pathological conditions, purity, dosage, and route of administration. For example, compared with those in healthy lean subjects, the levels of ET-1 in obese patients are generally elevated 29. ET-1 at 10-100 nM stimulates resistin and SOCS gene expression in adipocytes and induces vascular reactivity of the saphenous vein and internal mammary artery in healthy subjects and hyperfibrinogenemia patients 12, 13, 51. ET-1 dose-dependently stimulates proliferation (0.1-100 nM) and lipolysis (0.1-1000 nM) in murine preadipocytes 25, 36-39. ET-1 at 1-100 nM, but not 0.1 nM, decreases CD36 expression in vascular smooth muscle cells and adiponectin expression in 3T3-L1 adipocytes 36-39. Accordingly, the doses (0-100 nM) of ET-1 used in our study were selected on the basis of the literature and were in accordance with the effective doses (10-1000 nM) of ET-1 for regulating the expression of adipokines, adipogenesis, and inflammatory factors in fat cells 12, 13, 36-39. Strong conclusions as to whether any of these in vitro effects of ET-1 signals can help explain its in vivo effects on pathophysiological processes will require more thorough studies.

## Conclusions

In this study, ET-1 stimulated the growth of visceral HWPs via the ETAR, ETBR, ERK, JNK/c-JUN, JAK2/STAT3, AMPK, and PKC pathways but not the AKT or p38 MAPK pathways. Although neither ET-2 nor ET-3 altered the growth of visceral HWPs, the molecules targeted by ETAR and ETBR signaling to transduce ET-1 growth signals in HWPs may be similar (e.g., AMPK, PKC, and JAK2/STAT3) or different (e.g., ERK and JNK/c-JUN). However, the signaling pathways through which ETAR and ETBR control the ET-1-mediated growth of subcutaneous HWPs are seemingly similar. Targeting ET-1-stimulated signaling pathways may represent a promising therapeutic approach for managing obesity and associated metabolic disorders. Future investigations should prioritize the optimization of pathway-specific inhibitors, the elucidation of their systemic pharmacodynamic and pharmacokinetic effects, and the development of integrative therapeutic protocols to achieve long-term improvements in metabolic health.

## Supplementary Material

Supplementary figure.
