# Supplementary material for: Endothelin-1 Stimulates the Growth of Visceral and Subcutaneous Human Preadipocytes through Similar and Alternative Signaling Pathways via Type A and Type B Endothelin Receptors: Potential Implications for Therapeutic Strategies for Obesity and Metabolic Disorders
Source: Int J Med Sci. 2025 Oct 1;22(16):4161–74. doi: 10.7150/ijms.110073 (PMC12595340; doi:10.7150/ijms.110073)
Supplement: Supplementary file 1 — Supplementary figure. [file ijmsv22p4161s1.pdf]

Supplemental Figure 1. Differential expression of endothelin type A and type B receptors in different murine fat cell lines, such as 3T3-L1 white, C3H10T1/2 white, and HIB1B brown fat cells. However, neither receptor was expressed in other non-fat cell lines, such as H4IIEC hepatoma cells or 3T3 fibroblasts.

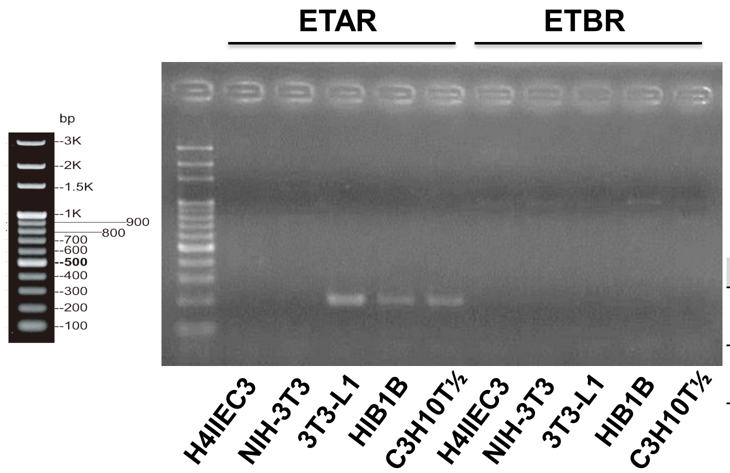

PCR condition (ETAR, ETBR)
95℃ 1 min > 57℃ 1 min > 72℃ 1 min, 0.5 μg template, 35 cycle

ETAR	Sequence (5'->3') length = 200
Forward primer	AAAGAAAGGCGTGAGACCAA
Reverse primer	GTGGTGCCCAGAAAGTTGAT

ETBR	Sequence (5'->3') length = 205
Forward primer	CGCTCTGTATTTGGTGAGCA
Reverse primer	AGTGAGATTCGGCGAGTGTT

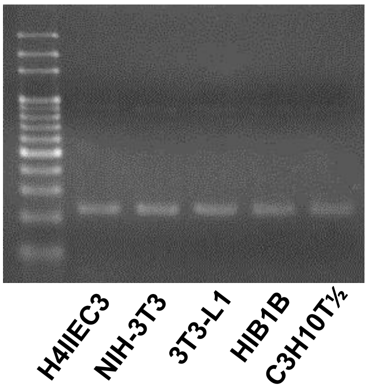

PCR condition (GAPDH)
95℃ 1 min > 62℃ 1 min > 72℃ 1 min 0.5 μg template, 21 cycle

GAPDH	Sequence (5'->3')
Forward primer	CCTCTGGAAAGCTGTGGCGT
Reverse primer	TTGGCAGGTTTCTCCAGGCG
